# Analysis of CDC social control measures using an agent-based simulation of an influenza epidemic in a city

**DOI:** 10.1186/1471-2334-11-199

**Published:** 2011-07-18

**Authors:** Yong Yang, Peter M Atkinson, Dick Ettema

**Affiliations:** 1 Department of Epidemiology, University of Michigan, Ann Arbor, 48109, USA; 2 Centre for Geographical Health Research, Geography and Environment, University of Southampton, Southampton, SO17 1BJ, UK; 3 Department of Human Geography and Planning, Utrecht University, Utrecht, 3508 TC, the Netherlands

## Abstract

**Background:**

The transmission of infectious disease amongst the human population is a complex process which requires advanced, often individual-based, models to capture the space-time details observed in reality.

**Methods:**

An Individual Space-Time Activity-based Model (ISTAM) was applied to simulate the effectiveness of non-pharmaceutical control measures including: (1) refraining from social activities, (2) school closure and (3) household quarantine, for a hypothetical influenza outbreak in an urban area.

**Results:**

Amongst the set of control measures tested, refraining from social activities with various compliance levels was relatively ineffective. Household quarantine was very effective, especially for the peak number of cases and total number of cases, with large differences between compliance levels. Household quarantine resulted in a decrease in the peak number of cases from more than 300 to around 158 for a 100% compliance level, a decrease of about 48.7%. The delay in the outbreak peak was about 3 to 17 days. The total number of cases decreased to a range of 3635-5403, that is, 63.7%-94.7% of the baseline value.

When coupling control measures, household quarantine together with school closure was the most effective strategy. The resulting space-time distribution of infection in different classes of activity bundles (AB) suggests that the epidemic outbreak is strengthened amongst children and then spread to adults. By sensitivity analysis, this study demonstrated that earlier implementation of control measures leads to greater efficacy. Also, for infectious diseases with larger basic reproduction number, the effectiveness of non-pharmaceutical measures was shown to be limited.

**Conclusions:**

Simulated results showed that household quarantine was the most effective control measure, while school closure and household quarantine implemented together achieved the greatest benefit. Agent-based models should be applied in the future to evaluate the efficacy of control measures for a range of disease outbreaks in a range of settings given sufficient information about the given case and knowledge about the transmission processes at a fine scale.

## Background

Since the beginning of the new millennium, epidemics of severe acute respiratory syndrome (SARS), avian influenza (bird flu) and H1N1 influenza (swine flu) have emerged repeatedly amongst the human population raising public concerns, particularly over future risk of disease, and underlying the need for increased understanding of transmission processes and the efficacy of alternative methods of control [1-9]. The transmission of an infectious disease amongst the human population is a complex process involving the time lines of infectious diseases, infection probability, contacts between individuals, demographic dynamics that determine the contact pattern and the occasional imported infection. At both the micro (for a single infection) and macro levels (the infection network) there exist a plethora of factors, objects and processes that combine to create complex scenarios, and this complexity varies from setting to setting.

Although several studies [10-12] have investigated the infection process directly by infecting healthy volunteers with the influenza virus in an experimental setting, for ethical reasons and because of the complexity involved, scientific experiments generally do not provide a feasible solution for the study of infectious disease transmission and the testing of control measures in a real world setting. This is especially true across whole communities or cities. It is, therefore, difficult to gain enough insight into the interplay of the various factors to predict the development of epidemics and formulate appropriate mitigation strategies. In these circumstances, modelling and simulation are potentially the most powerful tools available to increase understanding of infectious disease transmission processes and disease outbreaks. Importantly, modelling and simulation provide an important foundation for testing possible control measures [13].

It is difficult to build a satisfactory model of the transmission of infectious diseases due to deficiencies in both theory and data [14]. On the one hand, human knowledge of the infectious disease transmission process is limited at both the micro and macro levels. At the micro level, the exact process of a single infection for most types of infectious disease is unclear, even today [15]. At the macro level, disease diffusion amongst the human population is a complex process, as many factors, both social and physical, can contribute with different magnitudes to disease outcomes. On the other hand, it is difficult to obtain all the data required for model building, validation and simulation, especially for bottom-up approaches that require knowledge at the micro level.

In this paper, we argue that agent-based models (ABM) provide a powerful means to increase our understanding of infectious disease transmission. ABMs are computational models for simulating the actions and interactions of autonomous individuals within a heterogeneous population. ABMs are bottom-up models. That is, the macro-level behaviour of the whole system is generated by the simulation of the behaviour of agents at the micro-level. ABMs, formulated in different disciplines such as computer science [16], geography [17-20], epidemiology [21-25], and other interdisciplinary fields [26-28], can be applied to simulate the outbreak of infectious diseases and bio-terror attacks, and to explore the efficacy of control measures. The most important advantages of ABMs are that they can consider the heterogeneity of both individuals and environment, and also the stochastic essence of infectious disease transmission. ABMs can express explicitly the differences between individuals in terms of the attributes that influence the process of disease transmission such as physical, social, economic and environmental characteristics. For example, age, gender, occupation and lifestyle variables all contribute to the subsequent disease experience of an individual and the probability of infecting other individuals [29]. The interaction between individuals, which is one of the key components determining infectious disease transmission, can be expressed explicitly in the model. Using ABMs, the heterogeneity of the time lines of infectious diseases, infection probability, demographic dynamics that determine the contact pattern, and the occasional imported infection, can all be considered.

Influenza, an illness caused by ribonucleic acid (RNA) viruses that infect the respiratory tract, is transmitted mainly through the air by coughs or sneezes. Due to the high mutation rate of the virus, protection from any vaccine normally lasts only one year. In February 2007, the Centers for Disease Control and Prevention (CDC) in the United States issued the Community Strategy for Pandemic Influenza Mitigation [30] which is based upon an early, targeted, layered application of multiple partially effective non-pharmaceutical measures. Coupled with specific uses of antiviral influenza medications, these strategies aim to reduce transmission of pandemic influenza and mitigate the disease. Briefly, the main four interventions are: (1) isolation of influenza cases; (2) voluntary home quarantine of members of households with influenza; (3) school closure, which includes the dismissal of students from school (also including colleges, universities and childcare facilities) and school-based activities; (4) social distancing measures to reduce contact in the community and workplace. It also recommended that the intervention duration should be up to 12 weeks.

Amongst the four control interventions, the first two are straightforward and widely accepted by both the public and decision-making agents. The last two are controversial due to the high social and economic costs from closing schools and public places for certain periods, as well as the social and economic impacts of people refraining from their normal activities. Although non-pharmaceutical control measures are believed to be vital in curtailing the spread of disease, the quality of the evidence on which to base non-pharmaceutical pandemic planning decisions is poor [31]. Further, some research conclusions are contradictory. Take school closure as an example: although the importance of school pupils in the transmission of infectious diseases is acknowledged by most research [25,32-37], the effectiveness of school closure is not. As reviewed by the World Health Organization Writing Group [38] and Aledort et al. [31], although the majority of studies suggest the effectiveness of school closure, some previous studies showed that more cases developed after a school holiday, while schools that were kept open had a protective effect.

According to the Community Strategy for Pandemic Influenza Mitigation [30], the three major goals of mitigating a community-wide epidemic are: (1) delay the exponential growth in incident cases; (2) reduce the epidemic peak, and (3) reduce the total number of incident cases.

In this study, three properties of an epidemic outbreak were used to evaluate the effectiveness of a set of control measures. These were: (1) the total number of cases, (2) the number of cases at the peak and (3) the day number when the outbreak peaks.

This paper presents an analysis in which control measures are tested for a hypothetical influenza outbreak in Eemnes, a small city in the Netherlands, generated from the simulation of individuals' movements around the city and consequent interactions using a published ABM: Individual Space-Time Activity-based Model (ISTAM) [39,40]. ISTAM was applied to simulate an influenza outbreak for the same city in an earlier study, and the analysis included the spatial distribution of infection within the city, infection distribution at different types of locations and network analysis. This paper focuses on testing the CDC's suggested control measure strategies. This research provides new information which may be beneficial to the design of a control strategy for epidemic outbreaks, especially for some controversial intervention options such as school closure. Since the novel ABM framework, upon which the paper builds, was published previously by the authors, the basic structure and parameters of the model can be obtained from [32] and only a brief summary is given here. For the sake of brevity and clarity, the focus is on the efficacy of three of the CDC control measures.

The paper is organised as follows. The next section outlines the research methods. Then the simulation results and analysis are presented, followed by a section that discusses the merits and limitations of our approach. The last section draws conclusions regarding the application of ISTAM and remaining research issues.

## Methods

ISTAM is a bottom-up ABM in which the transmission network is built on the simulated physical contacts between individuals at a fine space-time scale [39,40]. At this scale, human social behaviour, the environment's physical conditions and the transmission mode of the specific infectious disease are considered. Activity bundle (AB) is a key concept in ISTAM. An AB is a semantic space where contact probability varies as a function of the dynamics of humans inside the bundle. The simulation is at two levels: people's movements between ABs and contact between individuals within ABs.

It is accepted that the possibility of infection for susceptible individuals increases with proximity to infectious individuals [41-43]. In addition, there exist social rules for how close humans can approach each other. Hall [44] identified four distances: intimate distance, personal distance, social distance and public distance. The values of the above distances vary between populations from different cultures, ages, genders etc. This knowledge provides a basis for within-AB simulation. For example, the model should focus on the changing distances between individuals and, specifically, when the distances are small enough for infection. The contacts between individuals are driven by social purposes or constrained by physical conditions or both, while infection *per se *is a physical process. Some contacts are indispensable for undertaking some activities. For example, an individual who goes shopping will generally make contact with the salesperson when checking out. Some contacts, although not purposeful, occur due to the restriction of the environment. For example, on a crowded bus, passengers must sit or stand in proximity. The first type of contact is voluntary and is determined by the individuals. The second type of contact is involuntary and is not wholly determined by the individuals.

The spatial distribution pattern of individuals can be observed in most types of AB. One example is provided by a restaurant: people sit in clusters which reflect the existence of different groups. Another example is provided by individuals visiting a library: people may try to find an empty table and sit as far as possible from each other [45]. In some ABs, such as in a lecture room, individuals are assumed to remain static during the simulation time unit. In other ABs, movements must be considered. At fine spatial scales, individuals' movement patterns may be strongly confined by the physical condition of the current AB and the status of other individuals. Three properties of humans' space-time dynamics within ABs that have been observed in reality are considered when modelling individuals within ISTAM: (1) individuals' static spatial distribution patterns, (2) individuals' movement patterns and (3) minimum distances between individuals. For more details about within-AB simulation using ISTAM, see [39].

ISTAM was previously applied to simulate hypothetical influenza outbreaks within the campus of the University of Southampton [40] and the city of Eemnes in the Netherlands [39]. The latter case provided an example of the simulation of the individual-based transmission of infectious disease at the whole city level. For the latter case, the first requirement was to build the population and the spatial structure of the city of Eemnes. The social and spatial structures of the city were built based on surveyed data of individuals' daily activities, synthesized data of households and land use data from previous research [46]. Properties such as family structure, number of cars and income level were assigned to every household and properties such as age and gender, and activity patterns, were assigned to every individual. Among the whole population of 8382 people, 3.2% were 4 years old or younger, 3.5% were between 5 and 9, 6.4% were between 10 and 17, 71.3% were between 17 and 64, and 15.6% were 65 years or older.

During the simulation, individuals' daily activities were generated from a distribution of observed activity patterns and then individuals' movements between ABs and the interaction of individuals within ABs were simulated. During these interactions, infection was possible conditional upon contact between individuals. For the latter case, parameters such as the contact frequency and contact intimacy which determine how often individuals come into contact and the probability for a contact to bring about infection in the model were calibrated by studies which indicated the average number of daily contacts for a person and the basic reproductive number for influenza *R*_0 _(the average number of secondary cases caused by each case of an infectious disease), respectively. In ISTAM, the latent period for influenza was assumed to be 1 to 3 days, the incubation period was one day longer than the latent period and the infectious period was 3 to 6 days [47-49]. The proximity and duration required for infection were assumed to be 1.5 m and 10 minutes, respectively.

A baseline simulation of a hypothetical disease outbreak in the city of Eemnes based on ISTAM was presented previously [39]. This paper extends the baseline model by testing three CDC control measures for a hypothetical influenza outbreak in Eemnes using ISTAM. These were:

(1) refrain from social activities (***A***): the probability of an individual to visit certain places such as social, leisure and sports facilities was decreased to a certain level;

(2) school closure (***S***): the probability of a pupil to visit school was decreased to 0;

(3) household quarantine (***H***): a certain proportion of individuals was required to stay at home at all times.

Because in reality, compliance to a control measure may be less than 100%, especially for the cessation of social activities and household quarantine, a series of compliance levels (25%, 50%, 75% and 100%) were tested for both ***A ***and ***H***. Two or three control measures can work together denoted by the abbreviations ***AS***, ***AH***, ***SH ***and ***SHA***, with a default 50% compliance level for both ***A ***and ***H ***and 100% compliance level for ***S***. The scenario with no control measures (denoted by ***N***) was also tested for comparison purposes.

An alert value (a threshold number of infection cases) was used to denote how quickly the control measure was implemented. At the beginning of each day, if the number of infection cases is greater than the alert value, the control measure will be implemented. This meant that there was a small delay of less than one whole day duration between exceeding the alert threshold and intervention. All control measures were assigned an alert value of 20 cases except ***N***. Additionally, for control measure ***SH***, a series of alert values (20, 50, 100, 200, 500 and 1000) and a series of rates of transmission (with *R*_0 _of 1.24, 1.79, 2.42, and 2.60) were explored. For each of the above scenarios, 300 simulations were run.

## Results

**Figure 1:**
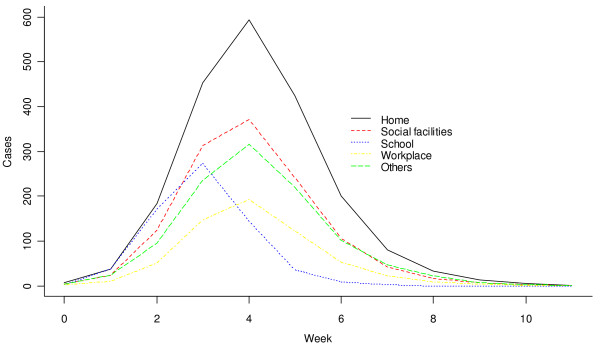
**Number of new cases from different ABs plotted against time (aggregated to units of a week) for the scenario ***N***, estimated over 300 simulations**.

Firstly, baseline simulations (***N***) were analyzed. Five individuals were selected randomly to be the index cases (the initial patients with influenza) and *R*_0 _was calibrated to be 1.79 [48]. Figure 1 shows the number of new cases from different ABs over time (in weeks). The three basic properties can be found in Table 1: on average, it took 31 days for the number of new infection cases to reach the peak, that is, 308 cases per day. The total number of cases during the outbreak was 5703.

**Table 1:** Total number of cases, peak number of cases and peak day under different control measures

Scenario ID	Compliance level	Probability of outbreak (%)	Total cases	Compared with *N *(%)	95% CI	Peak cases	Compared with *N *(%)	95% CI	Peak day	Compared with *N *(%)	95% CI
***N***		> 99	5703		5539-5872	308		274-345	31		22-44

	25%	99	5709	100.1	5522-5913	309	100.3	280-354	30	96.8	23-39
	
***A***	50%	100	5706	100.1	5537-5879	307	99.7	272-344	31	100.0	23-44
	
	75%	100	5701	100.0	5481-5921	307	99.7	264-346	31	100.0	23-44
	
	100%	98%	5695	99.9	5533-5890	308	100.0	271-349	32	103.2	23-51

***S***	100%	100	5461	95.8	5233-5666	219	71.1	187-256	39	125.8	30-51

	25%	100	5403	94.7	5138-5583	277	89.9	244-307	34	109.7	23-51
	
***H***	50%	93	4992	87.5	4758-5215	244	79.2	215-278	35	112.9	23-51
	
	75%	94	4428	77.6	4199-4704	204	66.2	178-243	41	132.3	23-65
	
	100%	68	3635	63.7	3376-3843	158	51.3	131-192	46	148.4	29-84

***AS***	50%, 100%	> 99	5449	95.5	5240-5648	222	72.1	192-253	39	125.8	30-51

***AH***	50%, 50%	97	5000	87.7	4754-5244	243	78.9	209-276	35	112.9	23-51

***HS***	50%, 100%	97	4494	78.8	4219-4781	134	43.5	107-160	48	154.8	36-71

***AHS***	50%, 50%, 100%	97	4453	78.1	4008-4753	135	43.8	104-162	51	164.5	36-79

Alert value is 20 for all scenarios except ***N***.

Table 2 shows the distribution of infection across different classes of AB. The four main infection sources were household (36%), social place (22.2%), workplace (10.9%) and school (11.9%). The peak day (i.e., day of the outbreak peak) of new infections was the fourth week for most types of AB (Figure 1), although, notably, was the third week for schools. Most infections at schools occurred from pupil to pupil. This implied that the epidemic outbreak was strengthened amongst children and then spread to adults. The control measure specific to schools may, therefore, be important, especially during the early stages of an outbreak.

**Table 2:** The proportion of infections between different types of AB using different control measures

	*N*	95% CI	%	*S*	95% CI	%	*H*	95% CI	%	*HS*	95% CI	%
Home	2049	1931-2137	36.0	2284	2197-2367	42.1	2129	2012-2246	42.7	2197	2043-2347	49.3

Work	618	570-660	10.9	630	580-678	11.6	478	430-524	9.6	469	422-529	10.5

Shops, post offices and banks	429	387-472	7.5	454	413-487	8.4	312	268-347	6.3	315	269-362	7.1

Healthcare facilities	493	458-538	8.7	536	481-580	9.9	359	325-404	7.2	385	345-432	8.6

Sports and cultural facilities	171	141-206	3.0	194	157-232	3.6	130	106-157	2.6	154	113-194	3.4

Social facilities	1253	1181-1339	22.0	1312	1224-1416	24.2	936	855-1016	18.8	926	856-1002	20.8

School	677	653-706	11.9	20	9-43	0.4	637	606-663	12.8	16	7-34	0.3

Alert value is 20 for all scenarios except ***N***; compliance level is 50% for ***H***.

**Figure 2:**
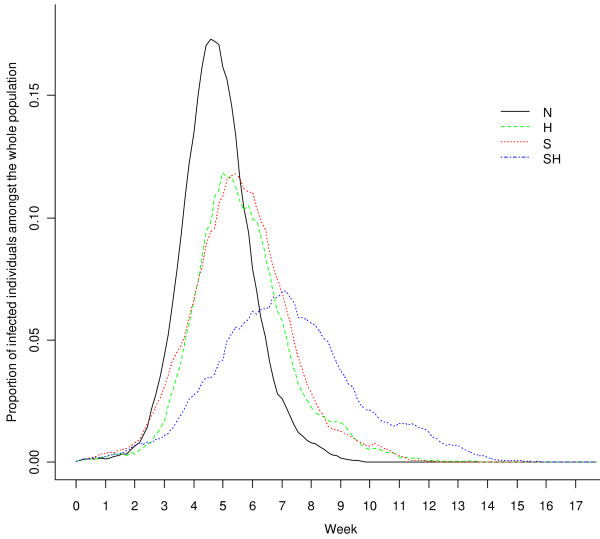
**Proportion of infected individuals amongst the whole population plotted against time (aggregated to units of a day) for the scenarios ***N***, ***S***, ***H ***and ***SH***, estimated by a selected typical simulation**.

For different control measures, the three properties of the outbreak under each scenario are given in Table 1. The proportion of infected individuals was plotted against time in Figure 2 for scenarios ***N***, ***S***, ***H ***and ***SH ***(for each scenario, the result of a "typical" simulation run is shown in which the three properties of the outbreak were similar to the average values for the 300 simulation runs). The implications from Table 1 and Figure 2 are:

(1) Amongst each single control measure, ***A ***was relatively ineffective. The values of all three properties for ***A ***were similar to those under the baseline scenario ***N***, with almost no variation with compliance level.

(2) ***H ***was a very effective control measure, especially in limiting the peak number of cases and total number of cases, with large differences between compliance levels. The decrease in the peak number of cases for ***H ***was from more than 300 to around 158 for a 100% compliance level, a decrease of about 48.7%. The delay in the peak day was about 3 to 17 days. Moreover, the total number of cases decreased to a range of 3635 - 5403, that is, 63.7% - 94.7% of the baseline value.

(3) The effectiveness of ***S ***was less than that of ***H***: the peak number of cases decreased to about 71.1% and the peak day was delayed by about 8 days. The effectiveness of ***S ***in reducing the total number of cases was small, a reduction of about 4.2%.

(4) For multiple control measures, since ***A ***had almost no effect, it is not surprising to see that ***AH ***was similar to ***H***, ***AS ***was similar to ***S ***and ***AHS ***was similar to ***HS***. ***HS ***(and ***AHS***) were the most effective control strategies: the peak number of cases decreased by 56% to 57%, the total number of cases decreased by 21% to 22% and the peak day was delayed by 17 to 20 days.

Table 2 lists the distribution of infections between different types of AB amongst ***S***, ***H ***and ***HS ***(***A ***is excluded due to its ineffectiveness). Compared with ***N***, ***S ***reduced the infection in schools to a very low level, but infection within other ABs also increased in absolute number, compensating for the gain. Compared with ***N***, ***H ***reduced the total infection, and the effectiveness varied amongst different types of AB. Thus, the transmission environments (ABs) most resistant to control were home and school, with decreases of 33% and 41%, respectively, while for all other ABs the total number of cases decreased by 63 - 69%. ***HS ***was the most effective control measure for all types of AB, with the lowest infection rates for any AB type.

Table 3 shows the three properties of the outbreak controlled by ***HS ***(with a 50% compliance level for household quarantine), with alert values increasing from 20 to 1000. Clearly, the higher the alert value, the larger the total number of cases and peak number of cases, and the earlier the peak day. With a lower alert value, that is, intervening earlier, control measures were more effective.

**Table 3:** Total number of cases, peak number of cases and peak day under ***HS ***control with various alert values

Alert value (infection cases)	Probability of outbreak (%)	Total cases	Compared with 20 (%)	95% CI	Peak cases	Compared with 20 (%)	95% CI	Peak day	Compared with 20 (%)	95% CI
20	97	4494		4219-4781	134		107-160	48		36-71

50	98	4503	100.2	4209-4832	142	106.0	119-174	38	79.2	23-51

100	98	4520	100.6	4152-4869	152	113.4	110-191	37	77.1	24-51

200	99	4586	102.0	4239-4839	173	129.1	139-208	31	64.6	23-44

500	97	4743	105.5	4454-5083	210	156.7	168-261	29	60.4	20-37

1000	97	4954	110.2	4752-5163	272	203.0	237-316	29	60.4	22-43

Compliance level is 50% for ***H***, 100% for ***S***.

Table 4 shows the three properties of the outbreak controlled by ***HS ***(with a 50% compliance level for household quarantine) for four values of *R*_0 _(1.24, 1.79, 2.42, and 2.60). By comparing the set of outcomes under control measure ***HS ***with the corresponding ***N ***scenario (for the same *R*_0_) (Table 4), it can be seen that the control measure ***HS ***was more effective for smaller *R*_0 _values. Because the smaller the *R*_0_, the lower the probability of an outbreak, the greater the relative decrease in total number of cases and peak number of cases, and the greater the relative delay in the peak day.

**Table 4:** Total number of cases, peak number of cases and peak day with or without ***HS ***control for four values of *R*_0_

*R*_0_ value	With *HS*?	Probability of outbreak (%)	Total cases	Compared with *N *(%)	95% CI	Peak cases	Compared with *N *(%)	95% CI	Peak day	Compared with *N *(%)	95% CI
1.236	No	80	3958		3575-4298	149		122-174	40		23-67
	
	Yes	41	2449	61.9%	895-2989	44	29.5%	17-51	94	235.0%	48-197

1.79	No	> 99	5703		5539-5872	308		274-345	31		22-44
	
	Yes	97	4494	78.8%	4219-4781	134	43.5%	107-160	48	154.8%	36-71

2.419	No	100	6955		6860-7066	503		460-561	25		22-30
	
	Yes	100	6299	90.6%	6142-6434	335	66.6%	293-380	34	128.0%	29-43

2.596	No	100	7522		7435-7585	658		607-720	22		19-26
	
	Yes	99	7154	95.1%	7063-7259	495	75.2%	461-539	28	127.3%	23-32

## Discussion

From the ISTAM model, school closure resulted in about a 4.2% reduction in the total number of cases and a 28.9% reduction in the peak number of cases. These reductions were rather low compared with some empirical studies [36,37] which suggest approximately a 15% reduction in the total number of cases and a 40% reduction in the peak number of cases. As Table 2 shows, with school closure, infection in schools was reduced to a very low level, but infection within other ABs increased in absolute number. The explanation is that school closure alone can move pupils' daily activities from school to home, and infection risk at home and other ABs may actually increase. This is consistent with other studies [31,32,50-53]. Milne et al. [51] argued in a comparison study that differences in effectiveness can arise from differences in assumptions about the timing and duration of school closure. It would be interesting to implement such finer level differences within the ISTAM model in future. Another explanation is that the current model does not consider the age dependence of susceptibility and infectiousness. For example, children may be more susceptible and infectious than adults [54,55]. This should be explored in the future.

This research highlighted the importance of combining control measures. As shown in Table 1, school closure alone was not particularly effective, but when combined with home quarantine, it reduced the total number of cases more than the sum of these two control measures alone. It should be noted that although the three control measures are applicable to different levels of aggregation (refraining from social activity is applicable to individuals, household quarantine is applicable to all members within households and school closure is applicable to all students), they are not independent of each other. For example, individuals under household quarantine automatically refrain from social activities, and students under household quarantine do not go to school. The overlap between control measures may explain the enhancing effect of combining measures. The combination of non-pharmaceutical measures with pharmaceutical measures needs to be investigated in the future, particularly as for infectious diseases with large *R*_0_, the effectiveness of non-pharmaceutical measures was shown to be limited.

ISTAM is a novel model for simulating the transmission of infectious disease. The two-level structure (separating between-AB and within-AB activities) makes ISTAM flexible such that it can be applied to novel circumstances. The concept of AB plays a key role: both the building of individual activity patterns and simulation within ABs depends on how well the ABs are defined and classified. The merits of simulation by ISTAM are: (1) ISTAM is straightforward and provides a process-based representation of the real world, (2) using ISTAM, it is easy to account for important factors, neglect less important factors and include random factors in the model and (3) ISTAM facilitates the modelling of human actions (both active and reactive) and the interaction between humans at fine scales. ISTAM, if fed with activity pattern data, can simulate effectively individuals' movements at between-AB and within-AB levels. Providing data sources are sufficient, the model can be extended readily to larger study areas although the effort involved in data collection and model implementation is likely to be substantial and time-consuming.

Despite the above advantages of ISTAM, the utility of the results presented here in terms of control measures depends on the ability of the ISTAM model to represent real-world processes such as human behaviour and movement patterns, both between and within ABs (generally buildings), the spatial structure of the set of ABs themselves, particularly at within-AB level, transmission probability as a function of the space-time separation between individuals, and the natural history of the targeted disease such as the time lines describing the evolution of infection and disease within the host (e.g., latent, infectious, incubation and symptomatic periods). For example, it is assumed that one requirement for transmission is a susceptible person within a certain distance of an infectious person. In fact, for most airborne infectious diseases, airborne disease agents may stay suspended in the air or survive on some surfaces such as door handles for an extended period of time. This suggests co-location may not be a strict requirement for infection. Applying an extended definition of effective contact will be a challenge for future research. Another problem arises due to limited validation. It was not possible to validate the model directly due to the lack of historical data on the impact of non-pharmaceutical control measures on disease outbreaks in Eemnes. Nevertheless, several strategies were implemented to validate the model indirectly [39], with satisfactory results.

The emergent space-time pattern of disease in a given region depends on the parameters of both the disease transmission model and the spatial and social network structures in place in the environment in which transmission takes place. In particular, it is expected that changes in the parameters of the (simulation) model will lead to observable changes in the space-time pattern of disease. Simulation models provide an important means for evaluating the sensitivity of emergent patterns and their space-time character to changes in model parameters. Thus, while the results of ABMs such as ISTAM depend on model parameterization, sensitivity analysis can be used to investigate the range of parameter values that leads to similar evidence for decision-making, providing reassurance to decision-makers.

If the association between elements of environmental and social structures and disease outcomes can be quantified then it should be possible to map the vulnerability of entire settlements to specific diseases. It is well-known that the behaviour characteristics of individuals can be modified to reduce the likelihood of disease transmission. However, the effects of spatial structural elements such as boarding school versus day school education for children and settlement structure (e.g., out-of-town supermarkets versus local shops) are less well studied. Again, such knowledge would be useful in terms of planning containment strategies and such knowledge can only be provided by simulation models such as ISTAM.

## Conclusions

ISTAM was used to quantify the efficacy of three of the social control measures recommended by the CDC. Such quantification, while carrying uncertainty due to model structure and parameterization, is not possible by other means such as *in vivo*, real life, epidemiological or biological experiments. Sensitivity analysis was used to explore the outcome of the simulation for a range of parameter values (compliance level, alert value, *R*_0_). For the simulated influenza outbreak in the city of Eemnes, the Netherlands, household quarantine was the most effective control measure in terms of the three indices including total number of cases, peak number of cases and peak day used to assess efficacy. Refraining from social activities was the least effective control measure. School closure alone was of limited efficacy, unless combined with other control measures such as household quarantine. When school closure was used singly, a proportion of infections were displaced to other settings. This conclusion has important implications and is consistent with other research [31,32,52,53].

ABMs should be applied in future to evaluate the efficacy of control measures for a range of disease outbreaks in a range of settings, conditional upon the availability of sufficient information about the scenario (e.g., demographics, the built environment and humans' daily activities) and knowledge about the transmission processes at a fine scale (specifically, the relationship between transmission and the space-time dynamics of individuals).

## Competing interests

The authors declare that they have no competing interests.

## Authors' contributions

All three authors contributed to the study design. YY implemented the model, analysed the results and drafted the paper. PMA took a key part in paper writing. DE provided the data sources and contributed to data preparation. All authors critically reviewed and revised versions of the manuscript.

## Pre-publication history

The pre-publication history for this paper can be accessed here:

http://www.biomedcentral.com/1471-2334/11/199/prepub
